# 11626 Impact of Preeclampsia on the Incidence of Breast Cancer in the Black Women’s Health Study

**DOI:** 10.1017/cts.2021.473

**Published:** 2021-03-30

**Authors:** Zahna R. Bigham

**Affiliations:** Tufts University

## Abstract

ABSTRACT IMPACT: This study will be the first to explore the relationship between preeclampsia and breast cancer risk using the largest cohort of Black women in the US, and it will guide future research and potentially inform clinical practice to reduce breast cancer disparities in this population. OBJECTIVES/GOALS: Black women are disproportionately impacted by preeclampsia. This disorder induces hormonal changes that may contribute to diseases such as breast cancer. However, there is a lack of clear data on the relationship between preeclampsia and breast cancer, and few previous studies included Black women. This study will work to fill this knowledge gap. METHODS/STUDY POPULATION: We prospectively assessed the association between preeclampsia during pregnancy and risk of breast cancer in 43,040 parous women in the Black Women’s Health Study, a nationwide cohort of Black women who were ages 21 -69 at enrollment in 1995. Through 2017, we confirmed 1,968 incident diagnoses of invasive breast cancer. Approximately 6% of parous women reported a diagnosis of preeclampsia; characteristics of the population at baseline are shown in Table 1. We used multivariable Cox proportional hazards models to estimate hazard ratios (HRs) and 95% confidence intervals (CIs) for risk of breast cancer overall. We used age as the time scale and adjusted for breast cancer risk factors including parity, age at first birth, age at menarche, and body mass index (BMI) at age 18. RESULTS/ANTICIPATED RESULTS: : Compared to parous women without a history of preeclampsia, women with a history of preeclampsia in any pregnancy were not at an increased risk of breast cancer overall (HR 0.98; 95% CI 0.81, 1.18). These preliminary results suggest that history of preeclampsia is not an important risk factor for breast cancer overall in Black women. Our analyses are ongoing to evaluate whether the association may vary by estrogen receptor status or within subgroups of the population defined by age, menopausal status, BMI, and time since last pregnancy. DISCUSSION/SIGNIFICANCE OF FINDINGS: Findings from this study will provide unprecedented knowledge on the association between hypertensive diseases during pregnancy and incidence of breast cancer in the largest cohort of Black women in the U.S.

